# Intraoperative Ex Vivo Confocal Microscopy for Surgical Margin Assessment in Squamous Cell and Basal Cell Carcinoma (HISTOBLOC): Protocol for a Prospective Monocentric Pilot Study

**DOI:** 10.2196/91436

**Published:** 2026-09-09

**Authors:** Giacomo Gravante, Agnès Leroux, Christian Garbar, Louise Henry, Sophie Cortese, Emilie Beulque, Romina Mastronicola, Gilles Dolivet

**Affiliations:** 1 Department of Surgical Oncology, Head and Neck Unit, Institut de Cancérologie de Lorraine, 6 Av. de Bourgogne, Vandœuvre-lès-Nancy, Grand Est, 54519, France, 33 03 83 59 85 66; 2 Department of Pathology, Institut de Cancérologie de Lorraine, Vandœuvre-lès-Nancy, Grand Est, France; 3 Faculty of Pharmacy, Université de Lorraine, Vandœuvre-lès-Nancy, Grand Est, France; 4 Faculty of Medicine, Université de Lorraine, Vandœuvre-lès-Nancy, Grand Est, France; 5 CRAN, CNRS, UMR 7039, Université de Lorraine, Vandœuvre-lès-Nancy, Grand Est, France; 6 Faculty of Dentistry, Université de Lorraine, Vandœuvre-lès-Nancy, Grand Est, France

**Keywords:** surgical margins, confocal microscopy, squamous cell carcinoma, basal cell carcinoma, intraoperative diagnosis, frozen sections

## Abstract

**Background:**

Accurate assessment of surgical margins is essential in the treatment of squamous cell carcinoma (SCC) of the upper aerodigestive tract or of cutaneous origin and of basal cell carcinoma (BCC). Intraoperative frozen section analysis is the current reference standard but is time-consuming and requires coordination between surgical and pathology teams. Ex vivo reflectance confocal microscopy offers rapid, real-time evaluation of surgical margins and may provide diagnostic information comparable to frozen section analysis while enabling the development of a reference atlas for tumor visualization.

**Objective:**

The HISTOBLOC study aims to evaluate the concordance between ex vivo confocal microscopy and intraoperative frozen section examination for assessing surgical margins in SCC and BCC. The secondary objectives are to assess diagnostic performance, time savings, clinical outcomes, and the feasibility of remote intraoperative evaluation using confocal microscopy.

**Methods:**

HISTOBLOC is a prospective, monocentric, single-arm pilot study conducted at the Institut de Cancérologie de Lorraine, a nonprofit comprehensive cancer center. The protocol plans on conducting a learning phase with 15 patients, followed by 30 consecutive patients, undergoing surgical excision for SCC or BCC (tumor size 1‐4 cm), whose margins are assessed with both ex vivo confocal microscopy and frozen section analysis; a minimum of four margin specimens are obtained per surgical procedure. The primary end point is the concordance (sensitivity, specificity, and positive and negative predictive values) between the two methods, with frozen section as the reference standard; the secondary end point is the time required for intraoperative margin assessment. Diagnostic accuracy is analyzed for the learning curve phase, for the post–learning curve phase, and for the overall study population.

**Results:**

Recruitment began on July 26, 2023, and ended on June 4, 2025. Statistical analysis and interpretation are ongoing. The final number of participants and of margin specimens included in the analysis, together with the study findings, will be reported in a separate publication.

**Conclusions:**

This protocol describes a study designed to determine whether ex vivo confocal microscopy can provide rapid, reliable intraoperative margin assessment comparable to frozen section analysis in head and neck and cutaneous SCC and BCC surgery.

## Introduction

### Background

Surgery remains the first-line treatment in nearly 80% of solid tumors, and achieving tumor-free surgical margins is critical for patient survival [1]. However, delineating the exact extent of a tumor is challenging, as surgeons rely on nonspecific visual changes and palpation. Margins must be sufficient to remove cancer cells while minimizing functional loss, and the recommended width varies by tumor type and risk category. For low-risk disease, margins of around 4 mm are typically used for basal cell carcinoma (BCC) and 4‐6 mm for cutaneous squamous cell carcinoma (SCC), whereas high-risk BCC and cutaneous SCC generally require wider margins, typically 6 mm or more (up to 10 mm), given their greater risk of subclinical extension [2-4]. For mucosal head and neck SCC, margins of 1 cm or more are generally recommended [5]. Small clusters of residual cancer cells or precancerous lesions often remain undetected, potentially leading to local recurrence or metastasis [1].

In BCC of the head and neck, the frequency of positive surgical margins ranges from 9% to 37.2% depending on tumor location and surgeon experience [6-10]. Large retrospective series have identified multifocality, Clark level, and depth of invasion as independent risk factors for incomplete excision [11]. Narrow margins (<4 mm) are associated with higher recurrence rates, with relapse occurring on average 31 months after surgery [12,13]. Accurate intraoperative detection of residual tumor cells is therefore essential. Conventional methods, such as frozen section analysis or touch imprint cytology, allow early detection but require significant time and coordination between surgical and pathology teams, prolonging procedures and potentially affecting patient management.

Reflectance confocal microscopy (RCM) has emerged as a rapid high-resolution technique (<1 mm) for ex vivo and intraoperative assessment of surgical margins, without interfering with subsequent histopathology [14]. In dermatologic surgery, RCM has demonstrated high sensitivity and specificity for BCC margins, with rapid image acquisition and evaluation [15,16]. Similar applications are under investigation in breast cancer, showing potential to reduce reoperation rates and improve intraoperative decision-making [17]. In eyelid and periocular tumor surgery, recent reviews have likewise highlighted confocal and related optical imaging as promising adjuncts to frozen section control [18]. These findings support RCM as a promising tool for real-time, accurate evaluation of surgical margins across multiple tumor types. Representative diagnostic performance figures from previously published studies are summarized in Textbox 1.

Textbox 1. **Diagnostic performance of ex vivo confocal microscopy reported in previously published studies.**

- Ex vivo confocal microscopy (Histolog V2) of 16 biopsies and 93 surgical specimens: processing time 5.1, SD 3.4 min; analysis time 1, SD 0.76 min; sensitivity 93% (biopsies) and 61.5% (surgical specimens); specificity 100% and 95%; BCC margin sensitivity 80%; and specificity 100% [15].
- Ex vivo confocal microscopy (Histolog V1) of 544 surgical samples from 148 patients with BCC: median time 5.17 min, sensitivity 73%, and specificity 96% [16].
- In 40 patients with breast cancer (POLARHIS study), surgeons and pathologists detected positive margins in 4 (33%) and 7 (58%) of 12 patients, respectively, using the Histolog scanner; these had not been detected by local intraoperative imaging techniques [17].

### Objectives

The primary objective of the HISTOBLOC study is to evaluate the concordance of intraoperative resection margin diagnosis based on ex vivo confocal microscopy images compared with standard frozen sections in patients with SCC of the upper aerodigestive tract, cutaneous SCC, or BCC.

The secondary objectives are the following:

- To assess the time saved by using confocal microscopy compared with standard frozen section examination during intraoperative evaluation of margin status
- To determine the concordance between confocal microscopy and the final histopathological examination
- To evaluate the rate of early local recurrence and the false-negative rate
- To assess the feasibility of a workflow based on rapid image acquisition and real-time transmission of confocal images to remote pathologists, enabling intraoperative pathological evaluation without the physical presence of the pathologist in the operating theater

We hypothesize that ex vivo confocal microscopy may provide margin diagnosis concordant with frozen section analysis while reducing intraoperative diagnostic time.

## Methods

### Study Design and Setting

HISTOBLOC is a prospective, monocentric, single-arm pilot study with minimal risks and constraints, conducted at the Institut de Cancérologie de Lorraine (ICL), a nonprofit comprehensive cancer center. The study is registered on ClinicalTrials.gov (NCT05935995).

### Ethical Considerations

#### Regulatory Requirements

The study is conducted in accordance with the ethical principles of the latest version of the Declaration of Helsinki and the Good Clinical Practice guidelines of the International Council for Harmonization (ICH-E6) [19]. It also complies with the applicable French regulations governing research involving human subjects, including the Data Protection Act, the Bioethics Act, and the decrees and orders implementing the Jardé framework [20-22].

#### Ethics Committee, Competent Authority, and Data Protection

Before conducting research on human participants, the sponsor submitted the project for review by a competent Committee for the Protection of Persons (CPP) and for information to the Agence nationale de sécurité du médicament et des produits de santé (ANSM). Requests for substantial amendments follow the same route. The study complies with the MR-001 reference methodology published by the Commission nationale de l’informatique et des libertés (CNIL).

#### Insurance

In accordance with the legislation in force under the French Public Health Code, the sponsor has taken out civil liability insurance covering the entire duration of the study and any participating party (policy B1339CTLICNWL22-74).

#### Patient Information and Informed Consent

Before enrollment in this minimal risk interventional research, participants provide free, informed, and express consent (written or oral) after being fully informed by the investigator during a consultation and after a sufficient reflection period. Participants may agree to the use of their data in future research for exclusively scientific purposes and may withdraw this consent or exercise their right to object at any time. If a protocol amendment revises the participant information and requires new consent, the request submitted to the CPP describes the procedures for obtaining it. The obligation to inform may be waived only when the person concerned cannot be located or when the competent CPP does not consider such information necessary (Article L.1123‐1) [23]. Personal information is kept in the participating physician’s file, treated as strictly confidential, and made available only to the competent authorities and duly authorized persons. In accordance with Article L.1122-1-1 [24], withdrawal of consent does not affect activities already carried out or the use of data obtained based on consent given before withdrawal.

#### Ethics Approval

The protocol was reviewed and received a favorable opinion from the OUEST III Committee for the Protection of Persons (Comité de Protection des Personnes OUEST III) on July 26, 2023 (reference 23.07.42). All participants provided informed consent as described above.

### Study Population

The HISTOBLOC study is offered to patients referred to the ICL for routine management of SCC of the upper aerodigestive tract or of cutaneous origin, or of BCC. Eligibility criteria are summarized in Textbox 2.

Textbox 2. **Inclusion and exclusion criteria.**

**Inclusion criteria**

- Age 18 years or older
- Tumor size 1-4 cm
- Tumor type: histologically confirmed squamous cell carcinoma (SCC) of the upper aerodigestive tract, cutaneous SCC, or basal cell carcinoma (BCC)
- Surgical indication validated by a multidisciplinary tumor board or by the treating surgeon
- Scheduled surgery
- World Health Organization (WHO) performance status ≤2
- American Society of Anesthesiologists (ASA) class ≤4
- Patient has understood, signed, and dated the informed consent form on the day of enrollment
- Patient affiliated with the social security system

**Exclusion criteria**

- History of irradiation to the surgical site
- Pregnant or breastfeeding women
- Individuals deprived of liberty or under legal guardianship (including conservatorship)

### Sample Size

The study is preceded by a learning phase involving 15 patients, during which participating surgeons are trained to interpret and analyze confocal microscopy images under the supervision of an experienced pathologist, to ensure adequate familiarization with the confocal features of surgical margins. After the learning phase, 30 consecutive patients are prospectively enrolled to provide a preliminary evaluation of the diagnostic concordance between confocal microscopy and intraoperative frozen section examination (the reference standard). Each surgical procedure yields a minimum of four surgical margin specimens. The planned sample size of 30 patients is justified based on the expected precision of the 95% CI estimates for the sensitivity and specificity of confocal microscopy in assessing margin status. Sample size calculations are performed assuming the minimum number of analyzable specimens (n=120), corresponding to the scenario yielding the widest (least precise) CIs, thereby providing a conservative estimation of diagnostic accuracy. These sample size calculations reflect the assumptions made at the design stage of the study and are independent of the final numbers of patients and specimens analyzed.

### Study Procedures

#### Information and Consent

Eligible patients receive detailed study information from an ICL surgeon during a preoperative consultation. After an appropriate reflection period and provision of free, informed, and written consent, eligibility criteria are verified. Included patients undergo surgical treatment in accordance with current recommendations specific to their pathology. Surgical margins are assessed using both the standard operating procedure (conventional histopathological examination, including intraoperative frozen sections) and the experimental operating procedure (ex vivo confocal microscopy using the Histolog scanner; SamanTree Medical, Lausanne, Switzerland).

#### Confocal Image Acquisition and Interpretation

After excision, each specimen is oriented with India ink and sectioned along its longitudinal axis; peripheral margin resections are obtained with a cold blade to avoid thermal artifact, and both sides of each margin are imaged. Specimens are then stained with Histolog Dip, an acridine orange-based solution that provides nuclear contrast without interfering with subsequent hematoxylin-eosin staining, and imaged ex vivo with the Histolog scanner using 488 nm laser excitation, with emission collected above 500 nm; each image is acquired in approximately 1 minute.

During the learning phase, confocal images are acquired and interpreted jointly by a pathologist and the surgeon. After the learning phase, confocal images are interpreted by the trained surgeon alone. At all times, confocal-based margin assessment is performed independently of, and blinded to, both the intraoperative frozen section result and the final histopathological examination, which serve as the reference standards.

#### Enrollment

Enrollment is carried out through the CleanWeb electronic case report form. A user guide is provided to investigators at site initiation. Access is secured for each user by an individual password-protected account; once inclusion is completed, an automatic email notification is sent to both the investigator and the sponsor.

#### Follow-Up and Premature Withdrawal

After completion of the surgical procedure, the patient exits the study and continues to receive clinical follow-up according to standard recommendations for their specific condition. Premature withdrawal may occur if surgery becomes definitively impossible, if the patient withdraws consent, or in case of loss to follow-up; if surgery is postponed, the patient remains in the study. Participants may withdraw consent and request removal from the trial at any time and for any reason, without justification and without affecting their right to standard medical care.

### Outcomes and Statistical Analysis

Quantitative variables are summarized using mean (SD), median, minimum and maximum values, and the first and third quartiles. Qualitative variables are summarized as absolute numbers and percentages.

To address the primary objective, the diagnostic accuracy of intraoperative surgical margin assessment using confocal microscopy is evaluated with frozen section histopathology as the reference standard:

- Sensitivity = true positives/(true positives + false negatives): the proportion of margins classified as positive by confocal microscopy that are confirmed positive by the reference standard
- Specificity = true negatives/(true negatives + false positives): the proportion of margins classified as negative by confocal microscopy that are confirmed negative by the reference standard
- Positive predictive value (PPV) = true positives/(true positives + false positives): the probability that a margin is truly positive when confocal microscopy yields a positive result
- Negative predictive value (NPV) = true negatives/(true negatives + false negatives): the probability that a margin is truly negative when confocal microscopy yields a negative result

Sensitivity, specificity, PPV, and NPV are reported as proportions with their 95% CIs, calculated on the total number of surgical margin specimens analyzed. To address the secondary objectives, the same diagnostic accuracy analyses are performed using the final histopathological examination as the reference standard for concordance. All diagnostic accuracy analyses are performed separately for the learning curve phase, the post–learning curve phase, and the overall study population.

The diagnostic time for confocal microscopy and for intraoperative frozen section examination, the time saved by confocal microscopy (the difference between these two diagnostic times), and the confocal processing time (including tissue resection and image acquisition and analysis) are summarized as described above. Diagnostic times obtained with the two methods are compared using a Student *t* test for normally distributed data or a Mann-Whitney *U* test otherwise; normality is assessed with the Shapiro-Wilk test.

The rate of early surgical recurrence potentially attributable to confocal microscopy is described by the false-positive rate (1 − specificity), corresponding to the proportion of histologically negative margins incorrectly classified as positive by confocal microscopy. The failure (nondetection) rate is described by the false-negative rate (1 − sensitivity), corresponding to the proportion of histologically positive margins incorrectly classified as negative. All analyses are performed in RStudio (version 2022.07.2+576; Posit PBC). The statistical significance threshold is set at *P* less than .05.

### Data Collection

**Figure 1.**
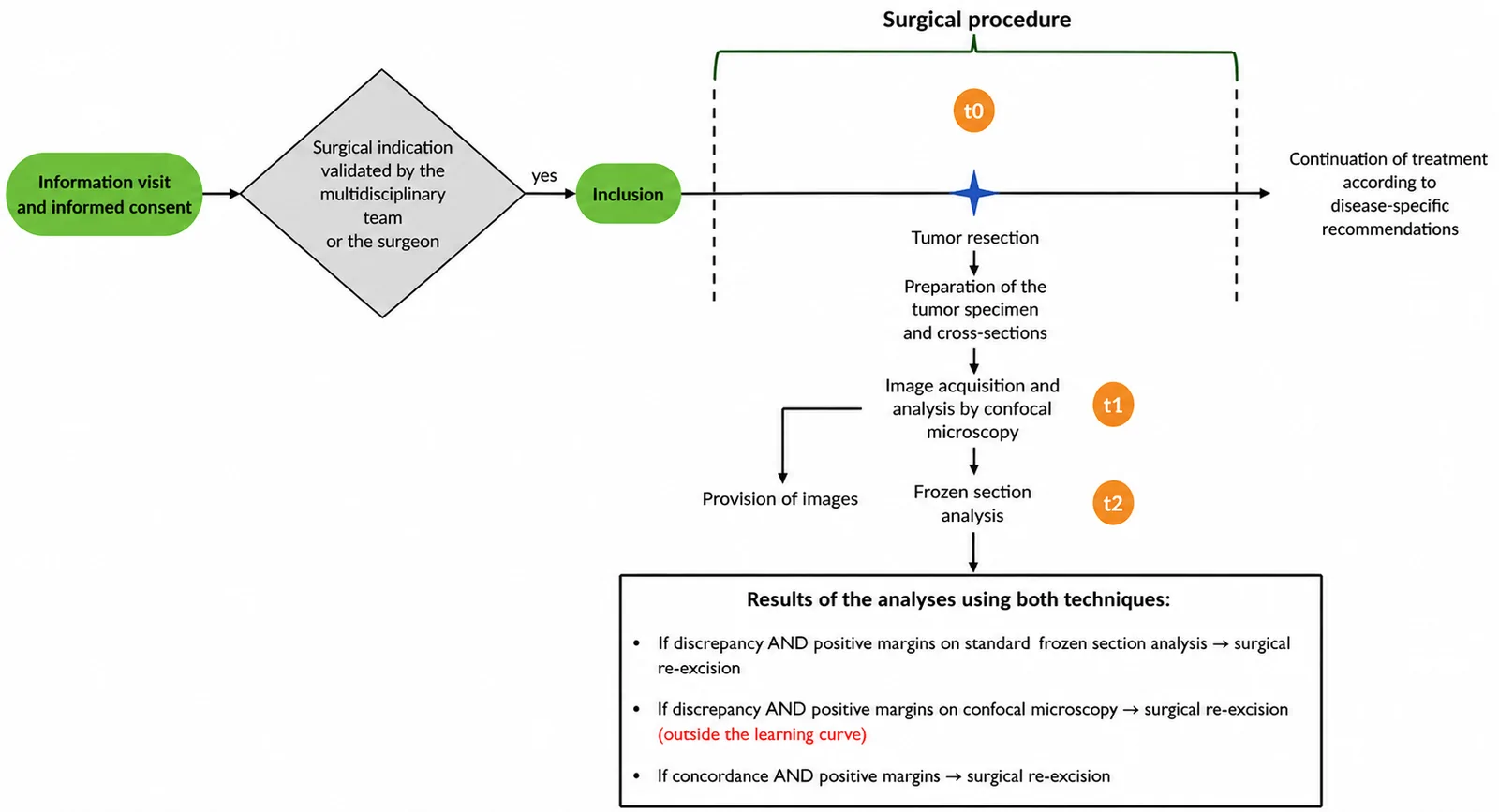
Timeline of the study. Legend: t0, t1, and t2 indicate the time of tumor removal, confocal image acquisition and analysis, and frozen section analysis, respectively. Surgical re-excision is performed when positive margins are identified by frozen section analysis (in all cases) or by confocal microscopy (outside the learning curve), or when the two techniques are concordant for positive margins.

Data collected in this study undergo a validation process to ensure completeness, authenticity, accuracy, and consistency; discrepancies are addressed through queries for clarification, and all data are stored and backed up on a dedicated secure server. Correction tables generated by the data management center are completed consistently by all personnel authorized to enter data into the case report form. Figure 1 shows the study timeline, and Table 1 summarizes the investigations and the data collected during the study.

**Table 1.** Summary of the investigations performed and data collected during the study.

	Inclusion	Surgery
Data collected during treatment		
General condition (WHO^a^ Performance Status)	✓	
Nutritional status	✓	
Medical history	✓	
Current weight and body shape	✓	
Height	✓	
BMI	✓	
Age	✓	
G8 score if patient 75 years or older	✓	
Usual treatment	✓	
Tobacco and alcohol consumption	✓	
Allergies	✓	
Extension assessment (scan and diagnostic biopsy)	✓	
Type of surgical procedure		✓
Initial diagnostic report	✓	
Surgical report		✓
Frozen section report		✓
Final pathology report		✓
Medical staff present in the operating room		✓
Data collected as part of the research		
Inclusion and exclusion criteria	✓	
Signed informed consent form	✓	
Confocal image analysis		✓
Tumor picture		✓
Analysis time record sheet		✓

a WHO: World Health Organization.

## Results

### Recruitment Status

Recruitment began on July 26, 2023, and ended on June 4, 2025. The final number of participants included and analyzed will be reported together with the study results in a subsequent publication.

### Planned Analyses

The diagnostic accuracy and time analyses described in the Methods are ongoing. Concordance metrics (sensitivity, specificity, PPV, and NPV with 95% CIs) and diagnostic time comparisons will be reported at the analysis levels, prespecified in the Methods, once statistical analysis and interpretation are complete.

### Dissemination Plan

Results will be disseminated through peer-reviewed publication and presentations at national and international otorhinolaryngology, head and neck surgery, and oncology meetings.

## Discussion

### Principal Findings

The HISTOBLOC study aims to prospectively evaluate the diagnostic accuracy of intraoperative ex vivo confocal microscopy for surgical margin assessment in head and neck and cutaneous carcinomas. Based on previous studies and clinical experience, positive surgical margins are expected in approximately 20% of cases [11,13]. Accordingly, diagnostic sensitivity and specificity will be estimated using 24 positive and 96 negative surgical specimens, respectively. Expected diagnostic performance assumptions are derived from dermatologic studies of BCC, in which confocal microscopy demonstrated an estimated sensitivity of 80% and specificity of 95% [15,16]. Under these assumptions, analysis of 96 specimens allows estimation of a lower 95% CI bound for specificity greater than 0.89 (range 0.89‐0.98), while analysis of 24 positive specimens allows estimation of a lower CI bound for sensitivity greater than 0.59 (range 0.59‐0.93), using the exact Clopper-Pearson method [25]. These estimates support the statistical robustness of the study design. The study will also establish whether confocal microscopy reduces intraoperative diagnostic time compared with frozen section analysis and will characterize diagnostic performance separately during and after the learning curve.

### Comparison With Prior Work

Previous evaluations of ex vivo confocal microscopy have focused primarily on dermatologic oncology and breast-conserving surgery, showing high diagnostic accuracy and potential workflow benefits [15-17], and recent narrative reviews have extended this interest to eyelid and periocular tumor surgery [18]. However, no prospective study has assessed this technique in head and neck oncologic surgery, where margin assessment is particularly challenging and clinically critical. HISTOBLOC extends prior work by applying confocal microscopy to SCC of the upper aerodigestive tract and related cutaneous malignancies, directly comparing it with intraoperative frozen section examination.

### Strengths

The main strengths of the study are its prospective design, the direct within-patient comparison of confocal microscopy with the intraoperative reference standard and with final histopathology, a structured learning phase to control operator dependence, a standardized ex vivo imaging protocol, and the concurrent development of a confocal reference atlas to support consistent interpretation and future training.

### Limitations

The limitations of this study include its single-center design and the operator-dependent nature of confocal image interpretation, which requires a structured learning curve. In addition, confocal microscopy primarily assesses surface margins and may be less sensitive to deeply infiltrative tumor growth, and the pilot sample size limits the precision of subgroup analyses. Finally, the number of patients and of margin specimens finally available for analysis may differ from the planned figures, which affects the precision of the estimates, in particular for the learning curve and subgroup analyses.

### Future Directions

If the pilot results are favorable, multicenter validation with a larger sample is warranted, together with formal evaluation of a remote real-time image transmission workflow for intraoperative tele-pathology, assessment of the impact of confocal-guided margin control on reoperation rates and clinical outcomes, and extension of the approach to additional tumor sites.

### Conclusions

HISTOBLOC is a prospective pilot study evaluating intraoperative ex vivo confocal microscopy in head and neck and cutaneous oncologic surgery. If high specificity, acceptable sensitivity, and meaningful time savings are confirmed, confocal microscopy may represent a valuable adjunct or alternative to frozen section examination, with the potential to reduce reoperations and optimize intraoperative decision-making. Within this framework, pathologists remain the cornerstone of pathological diagnosis, while confocal microscopy and digital image transmission are investigated as enabling technologies to enhance speed, accessibility, and intraoperative collaboration.

## Supplementary material

10.2196/91436 Checklist 1 SPIRIT checklist.

## Abbreviations

ANSM: Agence nationale de sécurité du médicament et des produits de santé (French National Agency for Medicines and Health Products Safety)
BCC: basal cell carcinoma
CNIL: Commission nationale de l'informatique et des libertés (French National Data Protection Commission)
CPP: Comité de protection des personnes (Committee for the Protection of Persons)
ICH: International Council for Harmonisation
ICL: Institut de Cancérologie de Lorraine
NPV: negative predictive value
PPV: positive predictive value
RCM: reflectance confocal microscopy
SCC: squamous cell carcinoma
